# M1CR0B1AL1Z3R 2.0: an enhanced web server for comparative analysis of bacterial genomes at scale

**DOI:** 10.1093/nar/gkaf413

**Published:** 2025-05-14

**Authors:** Yair Shimony, Edo Dotan, Elya Wygoda, Naama Wagner, Iris Lyubman, Noa Ecker, Gianna Durante, Gal Mishan, Jeff H Chang, Oren Avram, Tal Pupko

**Affiliations:** The Shmunis School of Biomedicine and Cancer Research, George S. Wise Faculty of Life Sciences, Tel Aviv University, Tel Aviv 69978, Israel; The Shmunis School of Biomedicine and Cancer Research, George S. Wise Faculty of Life Sciences, Tel Aviv University, Tel Aviv 69978, Israel; The Henry and Marilyn Taub Faculty of Computer Science, Technion—Israel Institute of Technology, Haifa 3200003, Israel; The Shmunis School of Biomedicine and Cancer Research, George S. Wise Faculty of Life Sciences, Tel Aviv University, Tel Aviv 69978, Israel; The Shmunis School of Biomedicine and Cancer Research, George S. Wise Faculty of Life Sciences, Tel Aviv University, Tel Aviv 69978, Israel; The Shmunis School of Biomedicine and Cancer Research, George S. Wise Faculty of Life Sciences, Tel Aviv University, Tel Aviv 69978, Israel; The Shmunis School of Biomedicine and Cancer Research, George S. Wise Faculty of Life Sciences, Tel Aviv University, Tel Aviv 69978, Israel; The Shmunis School of Biomedicine and Cancer Research, George S. Wise Faculty of Life Sciences, Tel Aviv University, Tel Aviv 69978, Israel; The Shmunis School of Biomedicine and Cancer Research, George S. Wise Faculty of Life Sciences, Tel Aviv University, Tel Aviv 69978, Israel; Department of Botany and Plant Pathology, Oregon State University, Corvallis, OR 97331, United States; Department of Computational Medicine, University of California Los Angeles, Los Angeles, CA 90095, United States; Department of Computer Science, University of California Los Angeles, Los Angeles, CA 90095, United States; Department of Anesthesiology and Perioperative Medicine, University of California Los Angeles, Los Angeles, CA 90095, United States; The Shmunis School of Biomedicine and Cancer Research, George S. Wise Faculty of Life Sciences, Tel Aviv University, Tel Aviv 69978, Israel

## Abstract

Large-scale analyses of bacterial genomic datasets contribute to the comprehensive characterization of complex microbial dynamics among different strains and species. Such analyses often include open reading frame extraction, orthogroup inference, phylogeny reconstruction, and functional annotation of proteins. We have previously developed the M1CR0B1AL1Z3R web server, a “one-stop shop” for conducting comparative analyses of microbial genomes. Here, we present M1CR0B1AL1Z3R 2.0, an enhanced version that includes a new user-friendly web interface and an improved, optimized, and more versatile pipeline. The following features were added: (i) a computationally efficient inference of orthogroups, which allows the analysis of up to 2000 bacterial genomes; (ii) genome completeness analysis; (iii) lists of orphan genes per genome; (iv) genome numeric representation that allows detecting genomic rearrangement events; (v) codon bias analysis; (vi) annotation of orthogroups with KEGG Orthology numbers; and (vii) a map of pairwise average nucleotide identity values. M1CR0B1AL1Z3R 2.0 is freely available at https://microbializer.tau.ac.il/.

## Graphical Abstract

**Graphical Abstract:**
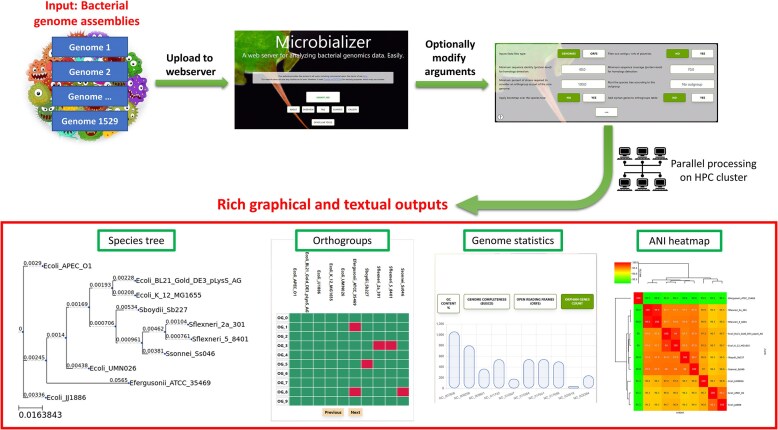

## Introduction

Next-generation sequencing (NGS) technologies are now routinely employed to sequence large collections of bacterial samples. Samples can include diverged species and strains or different isolates from a single species. The resulting sequence reads are assembled into contigs for each sample, forming a draft representation of the respective genome. These genomic assemblies are subsequently subjected to comparative genomics analyses, which highlight differences among the analyzed genomes and elucidate their phylogenomic relationships.

**Figure 1.**
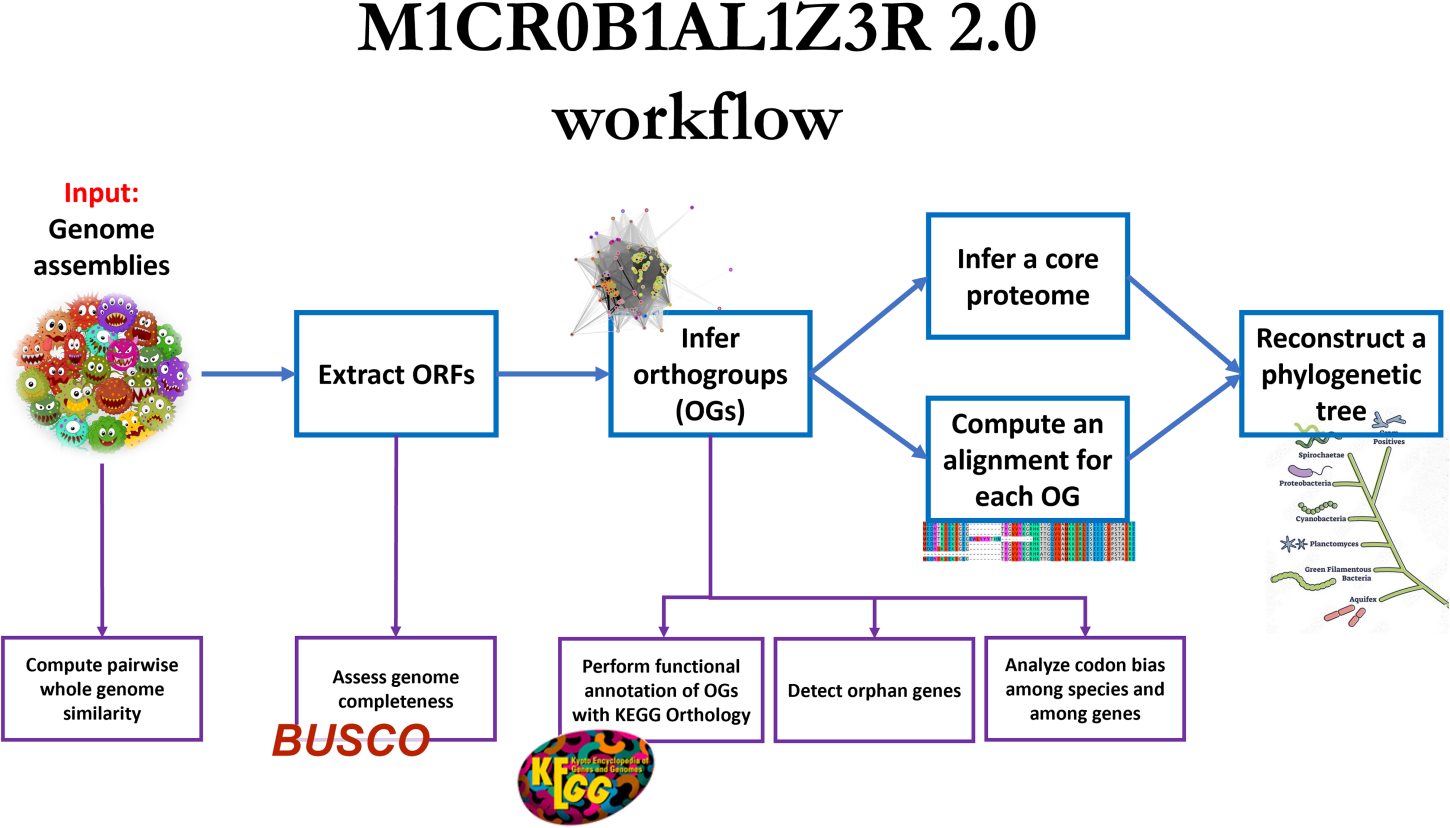
M1CR0B1AL1Z3R 2.0 pipeline workflow.

Common analyses include gene prediction, orthologous and paralogous relations prediction, phylogenetic inference, and functional annotation. Executing these computational tasks necessitates the integration of multiple bioinformatics tools. This complexity generally mandates the involvement of specialized bioinformaticians to design, implement, and maintain analysis pipelines. However, these pipelines frequently impose operational constraints, including specific software dependencies, high-performance computing (HPC) infrastructure (e.g. multicore servers), and advanced technical expertise for installation and execution. These limitations motivated the development of various ready-to-run pipelines and web servers to analyze datasets of microbial genomes [1–5]. We have previously developed M1CR0B1AL1Z3R [6] (pronounced: microbializer), a web-based platform designed to streamline microbial genome analysis and enhance accessibility for the broader scientific community. M1CR0B1AL1Z3R includes various analyses that are not provided by competing tools [6]. Here, we introduce M1CR0B1AL1Z3R 2.0, an enhanced platform featuring an updated pipeline (Fig. 1) that includes new outputs and algorithmic advancements, further enhancing the analysis of user-provided bacterial datasets with up to 2000 bacterial genomes.

## Materials and methods

In the subsequent sections, we detail the input of the pipeline, its processing steps, and the resulting output.

### Input

The pipeline input is a zipped folder with multiple FASTA-formatted files, each containing the genomic sequence of a single species/strain/isolate (we support up to 2000 genomes, a six-fold increase compared to the previous version). The file can contain a fully-assembled genome, a collection of contigs, or the set of open reading frames (ORFs) of a single genome. Notably, in many metagenomic studies, the assignment of the various contigs to separate isolates is unknown, and in this case, the data should be binned prior to running M1CR0B1AL1Z3R [7]. The input files should be provided as an archived (.zip or .tar.gz) folder.

An optional initial step in the pipeline involves filtering contigs or ORFs associated with plasmids by detecting the term “plasmid” in the record header. This filtering step can be activated via a user-defined flag.

### ORF extraction

We extract ORFs from each genome using Prodigal [8], as described in [6]. If a user uploads as input a dataset of DNA ORF files, this step is skipped. In both cases, the ORFs are translated into amino acid sequences, which are used in the next steps of the pipeline.

### Orthogroup inference

When considering a set of species, an orthogroup (OG) is defined as the group of genes descended from a single gene in the last common ancestor of the species. Orthogroup inference is the process of inferring all orthogroups in a dataset of genomes representing different species (or strains/isolates), i.e. clustering the (translated) ORFs of the genomes into orthogroups. Over the years, numerous computational methods have been developed for this purpose, beginning with Clusters of Orthologous Groups [9–13] and followed by algorithms such as InParanoid [14–16], OMA [17, 18], EggNOG [19, 20], OrthoMCL [21], and OrthoFinder [22, 23]. While each method defines its clustering objective slightly differently, the overarching goal is to infer groups of genes with orthologous relationships. In practice, this task is challenging due to gene duplication events, which introduce complex relationship patterns, including one-to-many and many-to-many orthologous connections. Additional complications stem from gene loss and lateral gene transfer events.

In this work, we implement a variant of the OrthoMCL method to cluster ORFs into groups comprising orthologs and recent paralogs [21]. The workflow begins with identifying reciprocal best hits (RBHs) between each pair of genomes [6] and recording the bit scores between protein RBHs. Sequence similarity searches are performed using MMseqs2 [24] with user-defined similarity and coverage thresholds. In parallel, paralogous gene pairs within each genome are identified under the same thresholds, retaining those with higher similarity to each other than to any ORF in another genome—these are classified as recent paralogs. Bit scores are subsequently normalized and ORFs are clustered using the Markov Cluster (MCL) algorithm [25], following the original OrthoMCL procedure. We adopt an inflation parameter of 1.5, as recommended by OrthoFinder [22], to balance cluster granularity and cohesiveness.

For large genomic datasets, the initial step of identifying RBHs between genome pairs becomes computationally prohibitive, as the number of pairwise comparisons increases quadratically with the number of genomes. To mitigate this limitation, and inspired by [4], we implemented the following optimization strategy. For datasets comprising a large number of genomes, we divide the input into smaller batches and infer orthogroups within each batch using the full algorithm described above. The output of this stage is a table of orthogroups for each batch. Subsequently, we construct a “pseudo-genome” from each such table by selecting a representative sequence from each orthogroup. The orthogroup inference algorithm is then reapplied to the set of pseudo-genomes. This step generates a table of orthogroups, each comprising sequences from pseudo-genomes. Finally, this table is updated by replacing each sequence within each orthogroup by the original sequences that it corresponds to (see Supplementary Material S1 for a detailed description).

The main output of this step is the orthogroup table, in which each row corresponds to an orthogroup and each column contains the set of genes from a specific genome. The $i,\ j$ entry contains the corresponding gene names of the ${{i}^{th}}$ orthogroup and ${{j}^{th}}$ genome. The orthogroup table is sorted by the ORF coordinates of the genome in the first column, i.e. the first orthogroup is the one that contains the first ORF of the genome in the first column. Orthogroups that do not contain a representative in the first genome are sorted by the ORF coordinates of the genome in the second column, and so on.

The pipeline provides several additional outputs that relate to the orthogroups table: (i) the orthogroup structure in OrthoXML format; (ii) a FASTA file encoding the phyletic pattern of the genomes and orthogroups, following the format described in [6]. The phyletic pattern constitutes a binary presence–absence matrix, where each genome is represented as a binary vector indicating its membership across orthogroups; (iii) a visual representation of this matrix as a blue–white grid, in which blue represents presence and white represents absence. The rows correspond to genomes and the columns to orthogroups. This matrix representation is enhanced with a dendrogram of the genomes; and (iv) the binary matrix is used to generate a two-dimensional UMAP projection [26] that groups together genomes with similar phyletic patterns. Additionally, the genomes are clustered using HDBSCAN [27], and each cluster in the UMAP is assigned a different color.

Following the orthogroup inference, we detect for each genome its orphan genes, i.e. genes belonging to that genome that do not have orthologs in any other genome. We distinguish between *orphan orthogroups*, which are clusters of paralogs in the genome that do not have orthologs in other genomes, and *orphan single genes*, which are genes in the genome that have neither orthologs nor paralogs.

### Alignments of orthogroups and phylogenetic tree reconstruction

For each orthogroup, all protein sequences are aligned using MAFFT with the “–auto” flag [28], and the aligned sequences are reverse-translated to generate codon-level alignments [29]. The multiple sequence alignments of all core orthogroups, i.e. orthogroups that contain an ORF from each genome, are concatenated to construct a core genome (using the DNA sequences) and a core proteome (using the amino acid sequences). In the case where there is more than one gene from a genome in a core orthogroup (e.g. due to gene duplication), we choose one randomly to be included in the concatenated core genome and proteome. To account for the absence of core orthogroups, users are given the option to adjust the threshold for the minimum percentage of genomes required for an orthogroup to be classified as core. For instance, if the threshold is set to 80%, orthogroups containing genes from at least 80% of the genomes will be considered core and included in the concatenated core genome and proteome.

A maximum-likelihood phylogenetic tree is reconstructed based on the concatenated core proteome, using IQ-Tree [30] with the WAG substitution model for protein evolution and rate heterogeneity modeled by a discrete Gamma distribution. A user can choose to compute branch support using bootstrap [31] and to root the tree by selecting one of the input genome names as an outgroup. If no outgroup is specified, the resulting Newick-formatted tree will be unrooted, whereas the graphical representation of the tree will be displayed using midpoint rooting. In cases where the concatenated core proteome contains >1000 core orthogroups, the species tree is reconstructed by randomly sampling 1000 core orthogroups, to reduce running times.

### Pairwise whole genome similarity

Average nucleotide identity (ANI) values are widely used for assessing pairwise whole-genome similarities [32–34]. We compute ANI values for each pair of genomes using FastANI [35] and represent the results as a heatmap to provide a visual representation of genome similarities. We also identify for each genome its closest relative. Notably, FastANI does not return results for genome pairs with ANI values significantly below 80%, leading to missing entries in the heatmap. When all genomes in the dataset exhibit relatively high similarity, resulting in no missing values, the heatmap is enhanced with hierarchical clustering to further illustrate genome relationships.

### Genome completeness

To evaluate genome completeness, we utilize the BUSCO framework [36]. This method uses a database of profile hidden Markov models (pHMMs) representing universal single-copy orthologs. A genome encoding ORFs with a significant match score against all these profiles is considered complete. Otherwise, the fraction of significant matches represents the level of completeness. We utilize the OrthoDB Bacteria dataset (version 9), which comprises 148 core-bacterial pHMMs [37]. Each input genome is queried against these profiles using *hmmsearch* from HMMER3 [38], and genome completeness is quantified as the fraction of profiles with at least one hit to a protein encoded in the genome, with an *E*-value threshold of 0.01.

### Genome numeric representation

**Figure 2.**
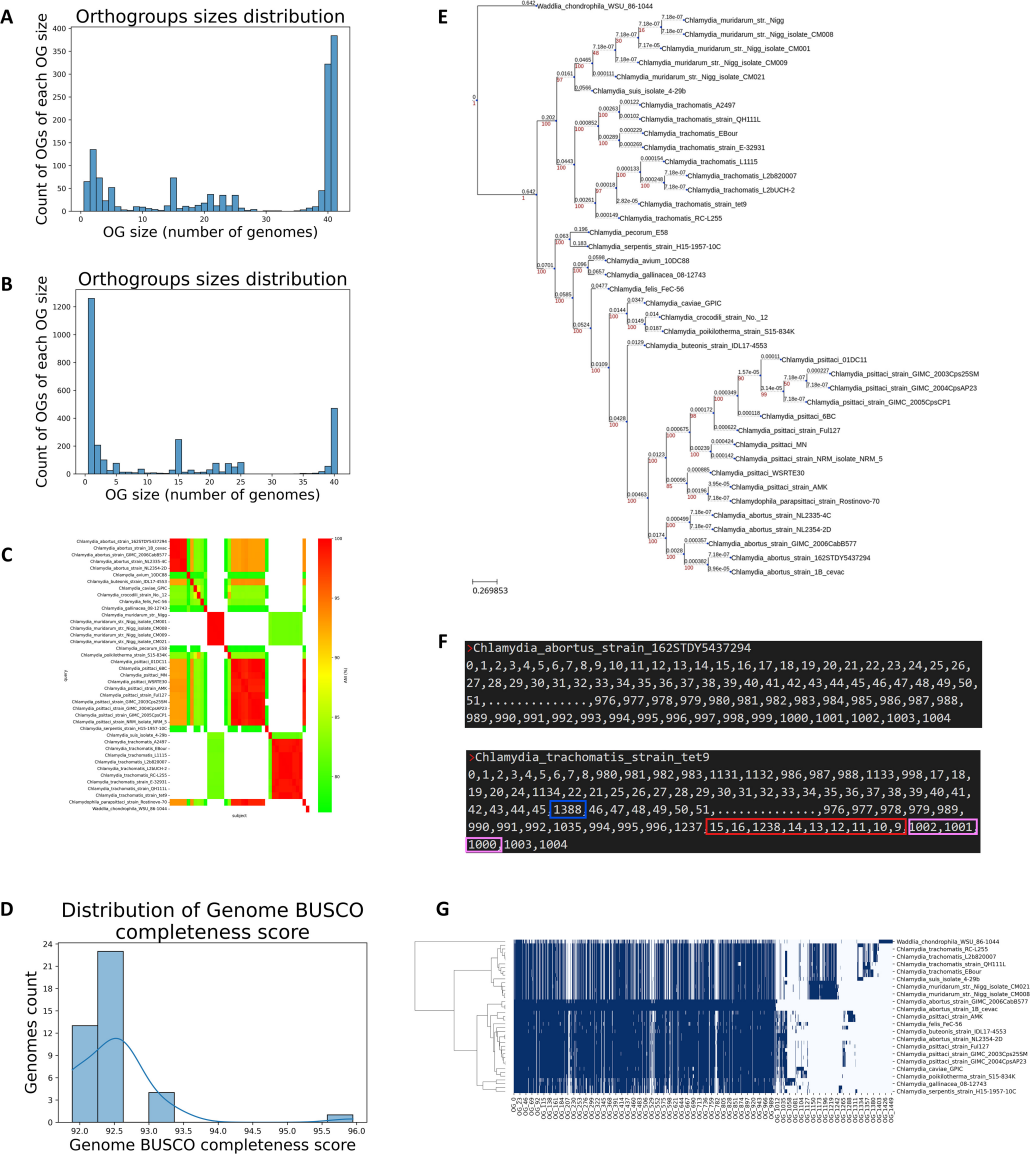
Comparative analyses of *Chlamydia* species using M1CR0B1AL1Z3R 2.0. (**A**) Histogram of orthogroup sizes from run A. (**B**) Histogram of orthogroup sizes from run B, demonstrating the effect of increased sequence identity and coverage thresholds on orthogroup formation. (**C**) Heatmap of ANI for the genomes analyzed in run A. The color scale represents ANI values, with red indicating high similarity and green denoting more distant relationships. (**D**) Distribution of genome BUSCO completeness scores from run A, showing the completeness of genome assemblies based on single-copy orthologs. (**E**) Phylogenetic tree reconstructed from run A, with *Waddlia chondrophila* used as an outgroup. Bootstrap support values are shown in red. (**F**) Genome numeric representation highlighting gene insertion (in blue), inversion (in pink), and translocation (in red) between *Chlamydia abortus* strain 162STDY5437294 and *Chlamydia trachomatis* strain tet9. The “...” is a placeholder representing all genes between #51 and #976. (**G**) Phyletic pattern of the genomes analyzed in run A, alongside a hierarchical clustering of them based on their orthogroup membership.

To identify genome rearrangement events, we perform an analysis termed “genome numeric representation.” This analysis begins with constructing for each genome a sorted list of its ORF identifiers. The list is sorted by the ORF coordinates in the genome. If the user uploads ORF files instead of full genomes, then we assume the order of the ORFs in each file corresponds to the genomic coordinates. Next, we replace each ORF identifier with the orthogroup number it belongs to or with the number −1 if it does not belong to any orthogroup. These numeric representations of all genomes are written to a single file, one row for each genome. This representation enables the straightforward visualization of genomic rearrangements, such as insertions, deletions, inversions, and translocations of genomic segments (Fig. 2).

### Functional annotation of orthogroups

KEGG Orthology (KO) annotations are assigned to each orthogroup using the approach described in KofamKOALA [39]. As a preprocessing step, the KOfam database (version 2024-09-01, based on KEGG release 111.0), which contains a pHMM for each KO along with a predefined score threshold, was downloaded and filtered to contain only prokaryotic-associated pHMMs. For an input dataset, rather than using the KofamScan tool [39], which we found to be computationally inefficient, we implemented an equivalent workflow that runs *hmmsearch* against the protein sequences of the orthogroups, using the profile database as a query. This is followed by filtering the results to retain only KO assignments with scores exceeding their respective thresholds. To optimize runtime, instead of analyzing all sequences within each orthogroup, we use the consensus sequence as a representative proxy (see Supplementary Material S2 for details).

### Codon bias analysis

Codon bias refers to the non-random usage of synonymous codons to encode a specific amino acid, a phenomenon widely observed across diverse organisms [40]. This bias is shaped by evolutionary forces, including selection for translational efficiency and accuracy, and is most pronounced in highly expressed genes (HEGs). Understanding codon bias is essential for investigating gene expression regulation and evolutionary dynamics at the genomic level. To quantify codon bias, as a preprocessing step, we generated an amino acid FASTA file comprising 40 well-characterized HEGs from *Escherichia coli*. The selection of these HEGs was based on data from CBDB [41], and the corresponding sequences were retrieved from the NCBI Protein Database.

For each analyzed genome, ORFs are screened for homologs of the HEGs using *tblastn* [42], where the genomic ORFs serve as the database, and the *E. coli* HEG protein sequences function as queries. Subsequently, the *W* vector of codon relative adaptiveness is calculated for each genome using the identified HEGs, following the method defined in [40], with Biopython [43] facilitating the computation. Additionally, genomes are clustered based on their *W* vectors, with principal component analysis used for visualizing the clusters.

In the next stage, the codon adaptation index (CAI), as defined in [40], is computed for all genes within each orthogroup, using the genome-specific *W* vector. The output includes the orthogroup table alongside the mean CAI for each orthogroup and a histogram depicting the distribution of mean CAI values across orthogroups.

### Outputs

Results are provided to the user as downloadable files when the pipeline finishes running. These files are organized in the following folders: (1) a table and heatmap of ANI values between all pairs of genomes; (2a) an ORF file of each genome; (2b) ORF count and GC content of ORFs for each genome, alongside matching histograms; (2c) an amino acid file for each genome that contains its translated ORFs; (3) BUSCO value of each genome alongside a matching histogram; (4) a list of orphan genes of each genome alongside statistics and a histogram; (5a) the inferred orthogroups in a table format, an annotated orthogroups table with orthogroup sizes, KO annotations, and the average CAI of each orthogroup, and the orthogroups in an OrthoXML format; (5b) a histogram depicting the distribution of orthogroup sizes (in terms of number of genomes included in each orthogroup), a phyletic pattern of the genomes and orthogroups, a matrix visualization of the phyletic pattern, a UMAP projection of the genomes (which are represented as binary vectors of orthogroup membership), a clustering of the genomes, and a numeric representation of the genomes; (6a) a FASTA file for each orthogroup with the unaligned DNA sequences; (6b) a FASTA file for each orthogroup with the unaligned amino acid sequences; (6c) a FASTA file for each orthogroup with the aligned amino acid sequences; (6d) a FASTA file for each orthogroup with the aligned DNA sequences (codon alignment); (7a) the concatenated core proteome and a list of the core orthogroups comprising it; (7b) the concatenated core genome and the same list of core orthogroups comprising it; (8) a species phylogeny tree in .newick, .png, and .svg formats; (9) a CSV file of the *W* vectors (codons relative adaptiveness) of all genomes, a clustering of the *W* vectors, the orthogroups table with the mean CAI value of each orthogroup, and a histogram of mean CAI values.

### Implementation

M1CR0B1AL1Z3R 2.0 is developed in Python 3.8, using the packages listed in https://github.com/orenavram/microbializer/blob/master/pipeline/microbializer.yaml. The web server operates on an HPC cluster hosted at Tel Aviv University, utilizing Slurm for job scheduling and resource management. To optimize runtime efficiency, each job’s computational steps are executed in parallel across multiple CPU cores and computing nodes. The web server includes a Gallery, an Overview, a Frequently Asked Questions (FAQ) section, and an output example, to assist users in maximizing the platform’s utility.

## Case study

To demonstrate the utility of M1CR0B1AL1Z3R 2.0 for comparative genomics, we analyzed a *Chlamydia* species genomes dataset. *Chlamydia* are intracellular bacterial pathogens that can infect various mucosal surfaces, leading to a range of health issues, especially in the reproductive and urinary systems [44]. Two independent analyses were conducted: run A, which included 40 *Chlamydia* genomes along with *Waddlia chondrophila* as an outgroup, and run B, which excluded the outgroup to focus exclusively on *Chlamydia* species. The analyzed genomes were downloaded from the NCBI repository in January 2025. The two runs also differed in their input parameters: in run A, we used the default thresholds for homolog detection (40% for sequence identity and 70% for sequence coverage), whereas in run B, we used a 60% threshold for sequence identity and 80% for sequence coverage. The complete results of these analyses are available in the Gallery section of the web server (https://microbializer.tau.ac.il/gallery).

Multiple genomic features were investigated (Fig. 2), including ORF counts, orthogroup distributions, ANI, orphan gene counts, genome completeness, and phylogenetic relationships. The core genome of *Chlamydia* (including the outgroup) was found to comprise 384 genes conserved across all strains. The ANI analysis revealed clustering patterns consistent with genetic divergence among *Chlamydia* species. Orthogroup analysis in run B, which employed stricter homolog detection thresholds, resulted in an increased number of orthogroups with fewer genes per orthogroup and a higher proportion of orphan genes. Genome completeness evaluations confirmed the high-quality assembly of most genomes, while phylogenetic analysis provided detailed insights into species relationships. Furthermore, genome numeric representation identified gene insertions, inversions, and translocations, highlighting structural genome variations across the analyzed species.

## Supplementary Material

gkaf413_Supplemental_File

## Data Availability

M1CR0B1AL1Z3R 2.0 is free and open to all users at https://microbializer.tau.ac.il/ and there is no login requirement. The source code of the pipeline, the pHMMs from OrthoDB v9 (utilized to assess genome completeness), and the FASTA file of *E. coli* HEGs (used for codon bias analysis) are available at https://github.com/orenavram/microbializer and https://doi.org/10.5281/zenodo.15306283.
