# Supplementary material for: M1CR0B1AL1Z3R 2.0: an enhanced web server for comparative analysis of bacterial genomes at scale
Source: Nucleic Acids Res. 2025 May 14;53(W1):W369–75. doi: 10.1093/nar/gkaf413 (PMC12230721; doi:10.1093/nar/gkaf413)
Supplement: gkaf413_Supplemental_File [file gkaf413_supplemental_file.pdf]

M1CR0B1AL1Z3R 2.0: an enhanced web server for comparative analysis of bacterial genomes at scale

Supplementary Material

AUTHORS

Yair Shimony[1,*], Edo Dotan[1,2], Elya Wygoda[1], Naama Wagner[1], Iris Lyubman[1], Noa Ecker[1], Gianna Durante[1], Gal Mishan[1], Jeff H. Chang[3], Oren Avram[4,5,6,*,†] and Tal Pupko[1,*,†]

[1]The Shmunis School of Biomedicine and Cancer Research, George S. Wise Faculty of Life Sciences, Tel Aviv University, Tel Aviv 69978, Israel
[2]The Henry and Marilyn Taub Faculty of Computer Science, Technion—Israel Institute of Technology, Haifa 3200003, Israel

[3]Department of Botany and Plant Pathology, Oregon State University, Corvallis, OR 97331, United States

[4]Department of Computational Medicine, University of California Los Angeles, Los Angeles, CA 90095, United States

[5]Department of Computer Science, University of California Los Angeles, Los Angeles, CA 90095, United States

[6]Department of Anesthesiology and Perioperative Medicine, University of California Los Angeles, Los Angeles, CA 90095, United States

[*]To whom correspondence should be addressed. Tal Pupko (Email: talp@tauex.tau.ac.il), Oren Avram (Email: orenavram@gmail.com), and Yair Shimony (Email: yairshsh@gmail.com).

[†]The last two authors should be regarded as Joint Last Authors.

Supplementary Material S1 – Orthogroup inference optimization

The primary computational bottleneck in orthogroup inference is the identification of reciprocal best hits (RBHs) between each pair of genomes, a process whose complexity scales quadratically with the number of genomes. To enable the analysis of large genome collections as a web service that receives multiple daily requests, we implement an optimization scheme inspired by the methodology proposed in panX (1).

The outline of this methodology is as follows: (a) genomes are partitioned into smaller batches; (b) The orthogroup inference algorithm is executed independently within each batch; (c) For each batch, a pseudo-genome is constructed by selecting a representative sequence from each inferred orthogroup; (d) The OrthoMCL algorithm is then applied to the set of pseudo-genomes, with a minor adjustment: within-genome homology searches (i.e., searching for paralogs) are omitted to reduce the likelihood of regrouping sequences that belong to different orthogroups within a batch. The output of this step is an orthogroups table of the pseudo-genomes; (e) Finally, all the representative sequences of the pseudo-genomes are replaced with the corresponding sets of original gene sequences, reconstructing the complete orthogroup structure.

For example, let's assume we have a dataset of 300 genomes, and a batch size of 10. Then we: (a) partition our genomes to 30 batches of 10 genomes each; (b) run OrthoMCL on each batch, generating 30 orthogroup tables, each with 10 columns; (c) convert each orthogroup table to a pseudo-genome by selecting a representative sequence from each orthogroup; (d) apply OrthoMCL again to the set of 30 pseudo-genomes (with the above-mentioned modification). We now have an orthogroup table of the pseudo-genomes, i.e., it has 30 columns; (e) replace each entry in this table (which is a sequence of a pseudo-genome) with the original sequences that it represents. Each entry represents sequences from up to 10 genomes. The final outcome is an orthogroup table of all 300 genomes, containing 300 columns.

We tested multiple variants of this optimization method to assess their impact on the final orthogroup output. Two datasets were used for benchmarking: dataset A, comprising 300 *Salmonella* strains and dataset B, containing 1,060 *Xanthomonas* and *E. coli* strains. NCBI accession numbers of the genomes in both datasets are available in

https://github.com/orenavram/microbializer. For each dataset, we executed the complete OrthoMCL algorithm as described in the main text, alongside various versions of the optimization method detailed above, to compare their performance and output consistency.

In the initial step, the genomes are partitioned into batches of size (i) 50, as implemented in panX, (ii) $\sqrt{n}$, where n denotes the total number of genomes, and (iii) $\sqrt[3]{2n}$, a value selected to minimize the number of pairwise comparisons in the RBH step. This optimal batch size was derived as follows: given a batch size of x, the number of comparisons within each batch is approximately x^2, resulting in a total of $x^2 \times \frac{n}{x}$ comparisons across all batches. Additionally, comparisons between the pseudo-genomes in the subsequent step contribute approximately $\left(\frac{n}{x}\right)^2$ comparisons. Therefore, the objective is to determine the value of x that minimizes the function:

$$f(x) = x^2 \times \frac{n}{x} + \left(\frac{n}{x}\right)^2 = nx + \left(\frac{n}{x}\right)^2$$

The minimum of this function is obtained when $x = \sqrt[3]{2n}$, which is the optimal batch size.

Additionally, for each of the three optimization variants, we evaluated two approaches for constructing the pseudo-genome: (a) selecting the first gene in each orthogroup as the representative sequence, and (b) using the consensus sequence of all genes within each orthogroup as the representative. The latter approach necessitates the multiple sequence alignment of all orthogroups within each batch, thereby increasing computational runtime.

For each of the six optimization variants, we evaluated the total runtime of the orthogroup inference step by summing all parallel computations. Of note, the reported runtime reflects the cumulative duration of all parallel computations. However, the actual wall-clock time experienced by the user is up to 50 times shorter. Since this user-perceived runtime is dependent on the availability of resources within our computing cluster and can vary between runs, we report the total aggregated runtime for consistency and reproducibility. We also recorded the number of orthogroups identified in the output table and assessed clustering accuracy by comparing the results to the orthogroups generated by the full OrthoMCL algorithm. Clustering accuracy was measured using five metrics from the *scikit-learn* package (2): *adjusted_rand_score*, *homogeneity*,

completeness, *v_measure* (three related metrics), and *fowlkes_mallows_score*. This analysis was conducted on dataset A (Table S1) and dataset B (Table S2).

The results presented in Tables S1 and S2 indicate negligible differences across all evaluated clustering metrics, suggesting that the optimization variants yield comparable outcomes. Consequently, we selected the variant with the shortest runtime, which involves partitioning the genomes into batches of size $\sqrt[3]{2n}$ and constructing the pseudo-genome by selecting the first gene in each orthogroup.

Table S1. Orthogroup inference optimization results for dataset A, comprising 300 *Salmonella* strains. *pseudo_genome_F* refers to the variant in which the first gene of each orthogroup is selected as the representative sequence for the pseudo-genome, while *pseudo_genome_C* refers to the variant in which the consensus sequence of each orthogroup is used as the representative. Clustering metrics are compared to the results of the full OrthoMCL algorithm, as described in the main text.

Orthogroup Inference Method	Aggregated Runtime	Number of orthogroups	Adjusted Rand Score	Homogeneity	Completeness	V measure	Fowlkes Mallows Score
original	23 days 22:18:31	23293	1	1	1	1	1
optimized – batchsize_50 pseudo_genome_F	4 days 12:34:33	23884	0.9955	0.9993	0.998	0.9986	0.9955
optimized - batchsize_$\sqrt[3]{2n}$ pseudo_genome_F	1 days 05:27:32	23404	0.9953	0.999	0.9984	0.9987	0.9953
optimized - batchsize_$\sqrt{n}$ pseudo_genome_F	1 days 16:24:35	23724	0.9945	0.9991	0.9979	0.9985	0.9945
optimized - batchsize_50 pseudo_genome_C	5 days 02:11:36	23974	0.9971	0.9993	0.9985	0.9989	0.9971
optimized - batchsize_$\sqrt[3]{2n}$ pseudo_genome_C	2 days 18:56:48	23599	0.9962	0.9992	0.9984	0.9988	0.9962

optimized - batchsize_$\sqrt{n}$ pseudo_genome_C	2 days 23:27:12	23914	0.9962	0.9993	0.9982	0.9987	0.9962

Table S2. Orthogroup inference optimization results for dataset B, comprising 1,060 *Xanthomonas* and *E. coli* strains.

Orthogroup Inference Method	Aggregated Runtime	Number of orthogroups	Adjusted Rand Score	Homogeneity	Completeness	V measure	Fowlkes Mallows Score
original	133 days 18:31:56	73814	1	1	1	1	1
optimized – batchsize_50 pseudo_genome_F	17 days 14:21:41	76575	0.9638	0.9959	0.9914	0.9937	0.964
optimized - batchsize_$\sqrt[3]{2n}$ pseudo_genome_F	10 days 17:30:57	74337	0.9658	0.9944	0.9929	0.9937	0.9658
optimized - batchsize_$\sqrt{n}$ pseudo_genome_F	15 days 18:19:10	76146	0.964	0.9956	0.9917	0.9936	0.9641
optimized - batchsize_50 pseudo_genome_C	19 days 19:36:54	77743	0.9686	0.9963	0.993	0.9946	0.9687
optimized - batchsize_$\sqrt[3]{2n}$ pseudo_genome_C	13 days 05:56:11	75411	0.9677	0.9949	0.9938	0.9943	0.9677
optimized - batchsize_$\sqrt{n}$ pseudo_genome_C	17 days 22:15:02	77249	0.9692	0.996	0.9931	0.9946	0.9693

Supplementary Material S2 – KO annotation optimization

To optimize the runtime of KEGG Orthology (KO) annotation, we investigated whether it is necessary to annotate all proteins in each orthogroup (OG) or if annotating a representative sequence and assuming that all other sequences within the OG have the same annotation would suffice. For benchmarking, we used dataset A from Supplementary Material 1 and tested three annotation variants: (i) annotating all proteins in each OG with KO numbers and unifying them to obtain a list of KOs assigned to the OG, (ii) annotating only the first protein in each OG, and (iii) calculating the consensus sequence of each OG and annotating it with KO numbers. The results of variants (ii) and (iii) were compared to variant (i), which served as the ground truth in this context. The results are summarized in Table S3. Notably, annotating only a single representative sequence per OG significantly reduced runtime, with the consensus-based annotation yielding better results. Consequently, we adopted the consensus approach in the pipeline.

Table S3. KO annotation optimization results for dataset A, comprising 300 *Salmonella* strains.

Optimization_mode	Precision	Recall	F1 score	Runtime (in hours, using 20 CPUs)
all_genes_of_og	1	1	1	12:10:48
first_gene_of_og	1	0.97	0.99	00:33:22
consensus_of_og	1	0.98	0.99	00:30:36

REFERENCES

1. Ding,W., Baumdicker,F. and Neher,R.A. (2018) panX: pan-genome analysis and exploration. *Nucleic Acids Res*, **46**, e5–e5.

https://doi.org/10.1093/NAR/GKX977

http://www.ncbi.nlm.nih.gov/pubmed/29077859

2. Pedregosa FABIANPEDREGOSA,F., Michel,V., Grisel OLIVIERGRISEL,O., Blondel,M., Prettenhofer,P., Weiss,R., Vanderplas,J., Cournapeau,D., Pedregosa,F., Varoquaux,G., *et al.* (2011) Scikit-learn: Machine Learning in Python. *Journal of Machine Learning Research*, **12**, 2825–2830.
